# Differences in the trophic ecology of micronekton driven by diel vertical migration

**DOI:** 10.1002/lno.11128

**Published:** 2019-02-04

**Authors:** Sonia Romero‐Romero, C. Anela Choy, Cecelia C. S. Hannides, Brian N. Popp, Jeffrey C. Drazen

**Affiliations:** ^1^ Department of Oceanography University of Hawaii at Manoa Honolulu Hawaii; ^2^ Integrative Oceanography Division Scripps Institution of Oceanography, University of California San Diego San Diego California; ^3^ Department of Earth Sciences University of Hawaii at Manoa Honolulu Hawaii

## Abstract

Many species of micronekton perform diel vertical migrations (DVMs), which ultimately contributes to carbon export to the deep sea. However, not all micronekton species perform DVM, and the nonmigrators, which are often understudied, have different energetic requirements that might be reflected in their trophic ecology. We analyze bulk tissue and whole animal stable nitrogen isotopic compositions (*δ* ^15^N values) of micronekton species collected seasonally between 0 and 1250 m depth to explore differences in the trophic ecology of vertically migrating and nonmigrating micronekton in the central North Pacific. Nonmigrating species exhibit depth‐related increases in *δ* ^15^N values mirroring their main prey, zooplankton. Higher variance in *δ* ^15^N values of bathypelagic species points to the increasing reliance of deeper dwelling micronekton on microbially reworked, very small suspended particles. Migrators have higher *δ* ^15^N values than nonmigrators inhabiting the epipelagic zone, suggesting the consumption of material during the day at depth, not only at night when they migrate closer to the surface. Migrating species also appear to eat larger prey and exhibit a higher range of variation in *δ* ^15^N values seasonally than nonmigrators, likely because of their higher energy needs. The dependence on material at depth enriched in ^15^N relative to surface particles is higher in migratory fish that ascend only to the lower epipelagic zone. Our results confirm that stark differences in the food habits and dietary sources of micronekton species are driven by vertical migrations.

Diel vertical migration (DVM) performed by marine zooplankton and micronekton is a ubiquitous behavior (Bianchi and Mislan 2016), constituting one of the largest animal migrations worldwide (Sutton 2013). Observations of higher biomass and abundance of organisms deeper in the water column during the day and closer to the surface at night have been extensively reported from global marine ecosystems (e.g., Vinogradov 1962; Clarke 1977; Pearcy et al. 1979; Watanabe et al. 1999). DVM is generally characterized by feeding in surface waters at night followed by excretion at greater depths, thereby contributing significantly to the transport of organic matter to the deep sea (reviewed in Drazen and Sutton [2017]). Bianchi et al. (2013) estimated that vertical migrators are responsible for between 15% and 40% of the total particle export flux to the mesopelagic zone. This active transport of carbon by DVM ultimately contributes to the functioning of deep‐sea ecosystems (Burd et al. 2010), and it is needed to meet the overall energetic demands of mesopelagic ecosystems (Steinberg et al. 2008; Robinson et al. 2010). Moreover, the transport of organic matter by vertical migrators is thought to enhance coupling between pelagic and benthic communities (Trueman et al. 2014) that in turn, improves the resilience of deep benthic systems to top–down or bottom–up perturbations that affect the structure of the food web (Blanchard et al. 2011).

Among vertical migrators, micronekton comprise much of the metazoan biomass in oceanic pelagic environments (Irigoien et al. 2014), yet the causes and implications of their migratory behavior are more poorly known than those of zooplankton (Lampert 1989; Hays 2003; Cohen and Forward Jr 2009). Micronekton comprise a taxonomically diverse group of organisms (i.e., fishes, crustaceans, and cephalopods) of ∼2 to 20 cm in length that are functionally defined as nekton (i.e., able to independently swim against currents). Many are zooplanktivores (Hopkins et al. 1997; Drazen and Sutton 2017) and are important prey for top predators, including commercially harvested species (e.g., Watanabe et al. 2009; Choy et al. 2013). Indeed a major driver of DVM is a response to avoid visual predators (Robison 2003), which adjust their behavior accordingly (Sainmont et al. 2013), further highlighting the implications of migrations for the trophic interactions within the food web. There are also nonmigrating species of micronekton which depend on food resources found at their specific habitat depth, likely resulting in different diet and trophic linkages compared with animals that migrate to access an array of food resources. However, nonmigratory micronekton are understudied (Klevjer et al. 2016), and it is unknown to what extent their different energetic requirements are reflected in their trophic ecology as compared to migrating species.

With energetic benefits exceeding the costs, DVM conveys an adaptive significance (Hays 2003). However, due largely to sampling constraints, the ultimate factor or the relative importance of the many drivers of DVM by micronekton is the subject of ongoing debate (Pearre 2003; Robison 2003). Past studies of stomach contents have revealed epipelagic zooplankton as the dominant prey of micronekton (Hopkins et al. 1997), as well as higher stomach fullness indices (Clarke 1977; Pearcy et al. 1979) or higher proportions of fresh food (Pearcy et al. 1979) in stomachs of individuals captured at shallower depths during the night. Collectively, these findings have led to the general assumption that vertically migrating micronekton feed at night near the surface and not during the day at depth (Robison 2003), following the movement patterns of zooplankton, their main prey. There is uncertainty associated with this interpretation because of net avoidance (Pearcy 1983), ingestion of food within nets (“net feeding”; Lancraft and Robison 1980), and stomach eversion of deep‐dwelling species. Hence, in order to understand the trophic implications of DVM, it is also necessary to trace diets at individual levels across longer time scales.

Stable isotope analyses have been widely used in studies of trophic ecology, providing a wealth of information about diet that is integrated over weeks or months (Vander Zanden et al. 2015). In particular, nitrogen isotope values (as *δ* ^15^N) increase ∼3‰ to 4‰ (Minagawa and Wada 1984) between a predator and its prey, representing an indicator of relative trophic position (TP). While a useful measure of TP, the *δ* ^15^N value of a metazoan can also vary depending on the *δ* ^15^N value of organic matter at the base of the food web. For example, the *δ* ^15^N values of suspended and sinking particulate organic matter are known to increase with depth due to microbial degradation (Saino and Hattori 1980; Casciotti et al. 2008). As a result, increases in *δ* ^15^N values with depth are also found in zooplankton (Koppelmann et al. 2009; Hannides et al. 2013) and deep‐benthic communities (Bergmann et al. 2009; Trueman et al. 2014). However, the depth‐related variation in *δ* ^15^N values has not been quantified for micronekton species (but see Choy et al. [2015] and Gloeckler et al. [2018] for amino acid compound‐specific isotope analysis [AA‐CSIA]). It is expected that nonmigrating species will show an increase in bulk tissue *δ* ^15^N values with depth, whereas if vertically migrating micronekton are feeding mainly in surface waters, they will exhibit similar *δ* ^15^N values to those of epipelagic nonmigrators, which in turn reflect the *δ* ^15^N values of surface plankton. Despite the potential of stable isotopic compositions to assess vertical migrations, this approach has been rarely used (Hannides et al. [2013] and Harris et al. [2014] in zooplankton and McClain‐Counts et al. [2017] in micronekton), and there is a lack of studies addressing differences in isotopic composition between migrators and nonmigrators along a depth gradient.

We compare the bulk tissue nitrogen isotopic composition between migratory and nonmigratory micronekton species inhabiting a depth range from 0 to 1250 m in the subtropical North Pacific. We aim to address the following questions: Do the *δ* ^15^N values of micronekton increase with depth of occurrence? Does feeding depth explain the vertical pattern in *δ* ^15^N values of both migratory and nonmigratory species? Is there a difference in the food habits and diet source between migrators and nonmigrators that can be inferred from nitrogen isotopic compositions?

## *Material and methods*

### Sample collection

The dataset was generated from a compilation of micronekton samples that were collected on different cruises that took place between 2007 and 2014 in February, March, April, May, July, or August at two sites to the north (Sta. ALOHA; 22.45°N, 158°W) and west (21.3°N, 158.3°W) of the island of O'ahu, and a third site near the island of Hawaii (Cross Seamount; 18.75°N, 158.25°W), all broadly within the central North Pacific Subtropical Gyre ecoregion (Supporting Information Fig. S1 and Table S1). Micronekton was sampled with tows of a 10‐m^2^ multiple opening closing net and environmental sensing system (MOCNESS; *see* Gloeckler et al. [2018] for details) or by oblique midwater trawls using a 3‐m‐Tucker Trawl (*see* Choy et al. [2015] for details), a Cobb Trawl (*see* De Forest and Drazen [2009] for details), or a Isaacs‐Kidd Midwater Trawl (IKMT; Supporting Information Table S1). Micronekton specimens were identified to species level (except for euphausiids, stomatopods, and *Vinciguerria* sp.), and lengths were measured onboard prior to storing them frozen at −80°C in cryovials. Samples were frozen individually, or as a pool of several individuals for small organisms where more tissue was needed for stable isotope analysis. Standard length, fork length, or total length measurements were taken for fishes, carapace length for crustaceans, and mantle length for cephalopods.

Samples of mesozooplankton (0.2 to > 5 mm) were collected in August 2011 (see Hannides et al. 2013 for details) at Sta. ALOHA and west of the island of O'ahu, and winter and summer 2014 at Sta. ALOHA. Zooplankton was sampled using a 1‐m^2^ MOCNESS net during the daytime (noon ± 2 h) and at nighttime (midnight ± 2 h) at depth intervals of: 0–50, 50–100, 100–150, 150–200, 200–300, 300–500, 500–700, 700–1000, and 1000–1500 m. Each mesozooplankton sample was size‐fractionated through 0.2, 0.5, 1.0, 2.0, and 5.0 mm mesh‐sieves and filtered onto 47‐mm filters of 0.2 mm nitex mesh, and stored frozen at −80°C.

### Sample processing and stable nitrogen isotope analysis

Each frozen sample was freeze dried, ground to a fine powder using pestle and mortar, and packed in 3.3 × 5 mm tin capsules. White muscle tissue was used for most of the micronekton species, but for specimens with insufficient muscle tissue, whole individuals were used after excising stomach contents. All sample preparation tools were rinsed with ethanol between each processed sample. Nitrogen isotopic composition was determined using an isotope ratio mass spectrometer (DeltaPlusXP) coupled to an elemental analyzer (Costech Model 4010). Isotopic ratios are given in *δ*‐notation as the deviation from the international standard, atmospheric N_2_, in parts per thousand (‰). Accuracy and precision were < 0.2‰ based on multiple analyses of glycine and homogenized fish tissue reference materials (both extensively characterized with NIST‐certified reference materials and their *δ* ^13^C and *δ* ^15^N values verified independently in other laboratories) and analyzed every 10 samples.

### Body size

Mass was used as an indicator of body size due to the large variability in morphologies of the species analyzed. Mass was measured in the laboratory on many frozen specimens prior to freeze drying (to within 0.01 g). For those specimens not weighed, an estimate of mass was obtained from body length using length/weight conversion factors available in the literature or calculated from weighed conspecifics from this study (Supporting Information Table S2).

### Data analysis

We used linear regression models to study the relationship between *δ* ^15^N values as the dependent variable and median depth of occurrence or log‐transformed body size as independent variables. Zooplankton *δ* ^15^N values of different years and seasons were averaged across depth intervals for night and day tows. To analyze the difference in *δ* ^15^N values between migrators and nonmigrators according to their depth of occurrence, we performed an analysis of covariance (ANCOVA), restricting the *δ* ^15^N values of nonmigrators for a depth range similar to that of migrators at nighttime (0–450 m). We evaluated seasonal variations within species by calculating the range between spring and summer mean *δ* ^15^N values for each species (i.e., the difference between mean spring and mean summer values). We then used a *t*‐test to determine potential significant differences between the seasonal ranges of migrator and nonmigrator values. We considered two seasons, spring (March and April) and summer (May, July, and August) according to annual variations in surface primary production and mixed layer depths at Sta. ALOHA (Karl et al. 2012). To disentangle the differences in the effects of depth and log‐transformed body size on *δ* ^15^N values between migrators and nonmigrators and a possible indirect effect due to the correlation between both predictors, we fitted a structural equation model (SEM; Grace 2006). All variables were standardized, followed by the calculation of the standardized path coefficients using the function *sem* of the package *lavaan* (Rosseel 2012). Those coefficients represent the effects of each predictor variable on the response variable in standard deviation units. The path diagrams were obtained from the function *semPaths* of the package *semPlot*. All analyses were performed using R (R Core Team 2015).

## *Results*

We determined *δ* ^15^N values of 20 nonmigrating and 25 migrating species of micronekton (*n* = 287) encompassing broad taxa (Actinopterygii, Malacostraca, and Cephalopoda). Individual masses ranged from 0.14 ± 0 g (mean ± SD, 49 ± 0 mm fork‐length) in the snake mackerel fish, *Gempylus serpens,* to 6.65 ± 0.53 g (73.92 ± 22.54 mm standard length) in the myctophid fish, *Bolinichthys distofax*. Median depth of occurrence for each species sampled, obtained from literature observations of their day and night ranges of distribution (Supporting Information Table S2), ranged from 0 to 1300 m for nonmigrators and from 150–895 to 0–382 m for migrators during the day and at night, respectively.

### Bulk *δ* ^15^N values of migratory and nonmigratory species

**Figure 1:**
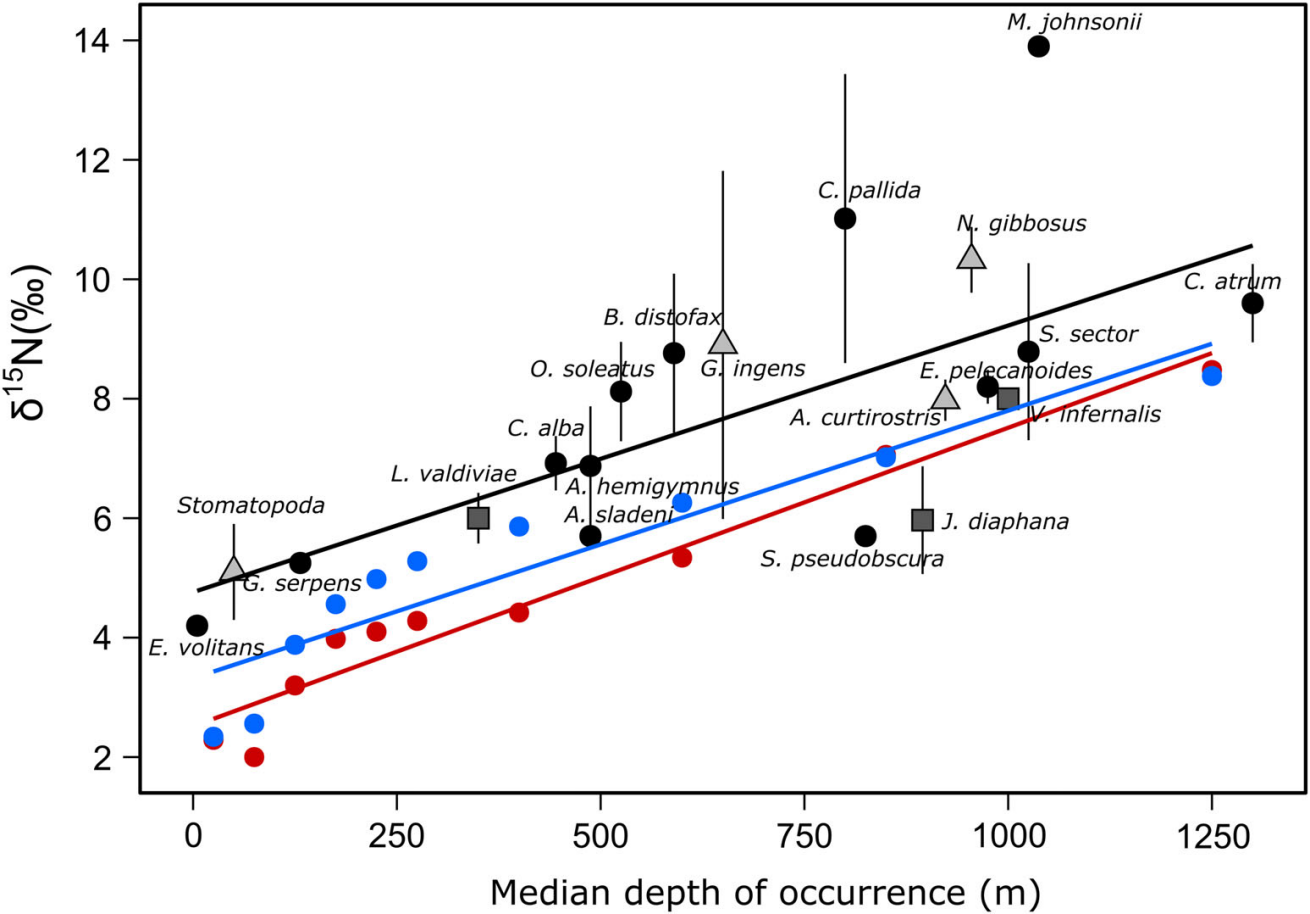
Relationship between *δ* ^15^N values (mean ± SD) and median depth of occurrence for nonmigrating micronekton species (black line; black circles, Actinopterygii; squares, Cephalopoda; and triangles, Malacostraca) values and between *δ* ^15^N values and median capture depth for zooplankton collected during the day (red line) and at nighttime (blue line). Zooplankton data are given as a mean for all size classes.

Nonmigratory species exhibited a significant increase in their *δ* ^15^N values with median depth of occurrence (*δ* ^15^N = 4.76 ± 0.85 + 0.0045 ± 0.001 × depth, parameter estimate ± SE, *R* ^2^ = 0.47, *p* < 0.001; Fig. 1). The *δ* ^15^N values of both day‐ and night‐collected zooplankton also showed the same increase with depth as the slopes of the *δ* ^15^N values vs. depth were not significantly different (ANCOVA *p* > 0.1). But for a given depth, the *δ* ^15^N values of all micronekton were significantly higher (ANCOVA *p* < 0.001) an average of 2.6‰ ± 0.8‰ (mean ± SD) than that of zooplankton. Zooplankton collected between 200 and 700 m at night had higher *δ* ^15^N values than zooplankton collected during the day within that depth range (Fig. 1).

**Table 1:** *δ* ^15^N values, body size (mean ± SD), and depth range of occurrence during the day and nighttime of the species analyzed in this study.

	Species	Class	*n*	Feeding guild	M/NM	Depth range day (m)	Depth range night (m)	*δ* ^15^N (‰)	Body size (g)
1	Euphausiidae	Mala	6	Sf	M	157	84	6.9±0.8	0.6±0.4
2	*Gennadas bouvieri*	Mala	6	Zoo	M	750–875	250–455	7±0.5	0.7±0.3
3	*Janicella spinicauda*	Mala	15	Zoo	M	400–600	0–200	5.7±0.8	0.5±0.3
4	*Oplophorus gracilirostris*	Mala	14	Zoo	M	490–650	60–200	6±1.1	1.4±0.5
5	*Sergestes erectus*	Mala	7	Zoo	M	550–800	250–325	6.1±0.8	0.9±0.1
6	*Sergia gardineri*	Mala	5	Zoo	M	650–775	0–150	4.5±0.2	0.2±0.3
7	*Systellaspis debilis*	Mala	3	Zoo	M	550–800	100–300	6.7±1.2	1.5±0.1
8	*Abralia trigonura*	Cepha	6	Zoo	M	390–650	30–200	6.7±0.9	2.7±0.3
9	*Abraliopsis* sp*. A*	Cepha	5	Zoo	M	475–700	20–200	7.4±0.5	2.7±0.4
10	*Hyaloteuthis pelagica*	Cepha	11	Zoo	M	100–200	0–50	7.7±0.2	1.7±0.3
11	*Pterygioteuthis microlampas*	Cepha	6	Zoo	M	450–575	25–180	5.4±1	0.3±0.5
12	*Benthosema suborbitale*	Actino	4	Zoo	M	490–620	15–75	6.1±0.7	0.3±0.3
13	*Bolinichthys longipes*	Actino	15	Zoo	M	525–725	50–150	7±1.2	0.9±0.3
14	*Ceratoscopelus warmingii*	Actino	20	Zoo	M	600–1000	0–150	6.2±1.3	1.9±0.2
15	*Chauliodus sloani*	Actino	8	Micro	M	450–825	45–225	7.2±1.1	3.7±0.7
16	*Diaphus perspicillatus*	Actino	8	Zoo	M	490–560	30–190	7.4±0.7	2.1±0.3
17	*Eustomias bifilis*	Actino	2	Micro	M	650–800	15–200	4.8±0.1	0.9±0.1
18	*Gonostoma atlanticum*	Actino	3	Zoo	M	490–560	150–300	8.7±0.6	1.1±0.1
19	*Hygophum proximum*	Actino	7	Zoo	M	500–700	25–150	5.5±1.2	0.7±0.5
20	*Idiacanthus fasciola*	Actino	9	Micro	M	550–800	0–300	7.7±1.3	2.7±0.4
21	*Lampanyctus nobilis*	Actino	9	Zoo	M	590–1200	40–140	7.6±1.3	2.1±0.3
22	*Myctophum lychnobium*	Actino	2	Zoo	M	600–800	0–15	5.4±1	0.7±0.7
23	*Lampanyctus niger*	Actino	14	Zoo	M	640–900	100–310	8.9±1.1	2.5±0.3
24	*Nealotus tripes*	Actino	8	Micro	M		50–200	6±1.1	0.2±0.2
25	*Vinciguerria* sp.	Actino	5	Zoo	M	400–560	20–125	7.9±0.5	0.7±0.1
26	*Acanthephyra curtirostris*	Mala	8	Zoo	NM	700–1500	500–1250	8±0.3	1.4±0.3
27	*Gnathophausia ingens*	Mala	3	Zoo	NM	400–900	400–900	8.9±2.9	2.5±0.6
28	*Notostomus gibbosus*	Mala	4	Zoo	NM	600–1500	600–1500	10.3±0.6	2.7±0.3
29	Stomatopoda	Mala	8	Zoo	NM	0–100	0–100	5.1±0.8	0.2±0.4
30	*Japetella diaphana*	Cepha	3	Zoo	NM	725–1065	725–1065	6±0.9	6.5±0.1
31	*Liocranchia valdiviae*	Cepha	2	Zoo	NM	0–700	0–700	6±0.4	0.3±0.4
32	*Vampyroteuthis infernalis*	Cepha	1	Zoo	NM	800–1200	800–1200	8	0.6
33	*Argyropelecus hemigymnus*	Actino	4	Zoo	NM	425–550	425–550	6.9±1	0.3±0.3
34	*Argyropelecus sladeni*	Actino	1	Zoo	NM	400–575	400–575	5.7	1.1
35	*Bolinichthys distofax*	Actino	8	Zoo	NM	490–690	490–690	8.8±1.3	6.7±0.5
36	*Cyclothone alba*	Actino	5	Zoo	NM	425–465	425–465	6.9±0.5	0.2±0.5
37	*Cyclothone pallida*	Actino	18	Zoo	NM	600–1000	600–1000	11±2.4	0.4±0.4
38	*Cyema atrum*	Actino	3	Zoo	NM	1200–1400	1200–1400	9.6±0.7	4.1±0.1
39	*Eurypharynx pelecanoides*	Actino	2	Zoo	NM	650–1300	650–1300	8.2±0.3	2.1±0.1
40	*Exocoetus volitans*	Actino	1	Zoo	NM	0–10	0–10	4.2	0.9
41	*Gempylus serpens*	Actino	2	Zoo	NM	44–219	44–219	5.3±0.1	0.1±0
42	*Melanocetus johnsonii*	Actino	2	Micro	NM	850–1225	850–1225	13.9	1.9±0.4
43	*Opisthoproctus soleatus*	Actino	5	Zoo	NM	450–600	450–600	8.1±0.8	5.4±0.1
44	*Serrivomer sector*	Actino	8	Micro	NM	550–1500	550–1500	8.8±1.5	4.1±0.3
45	*Sternoptyx pseudobscura*	Actino	1	Zoo	NM	725–925	725–925	5.7	0.5

Class: Cepha, Cephalopoda; Mala, Malacostraca; Actino, Actinopterygii. n, number of samples. Feeding guild: Zoo, zooplanktivore; Micro, micronektonivore; Sf, suspension feeder. M/NM: M, migrator; NM, nonmigrator.

**Figure 2:**
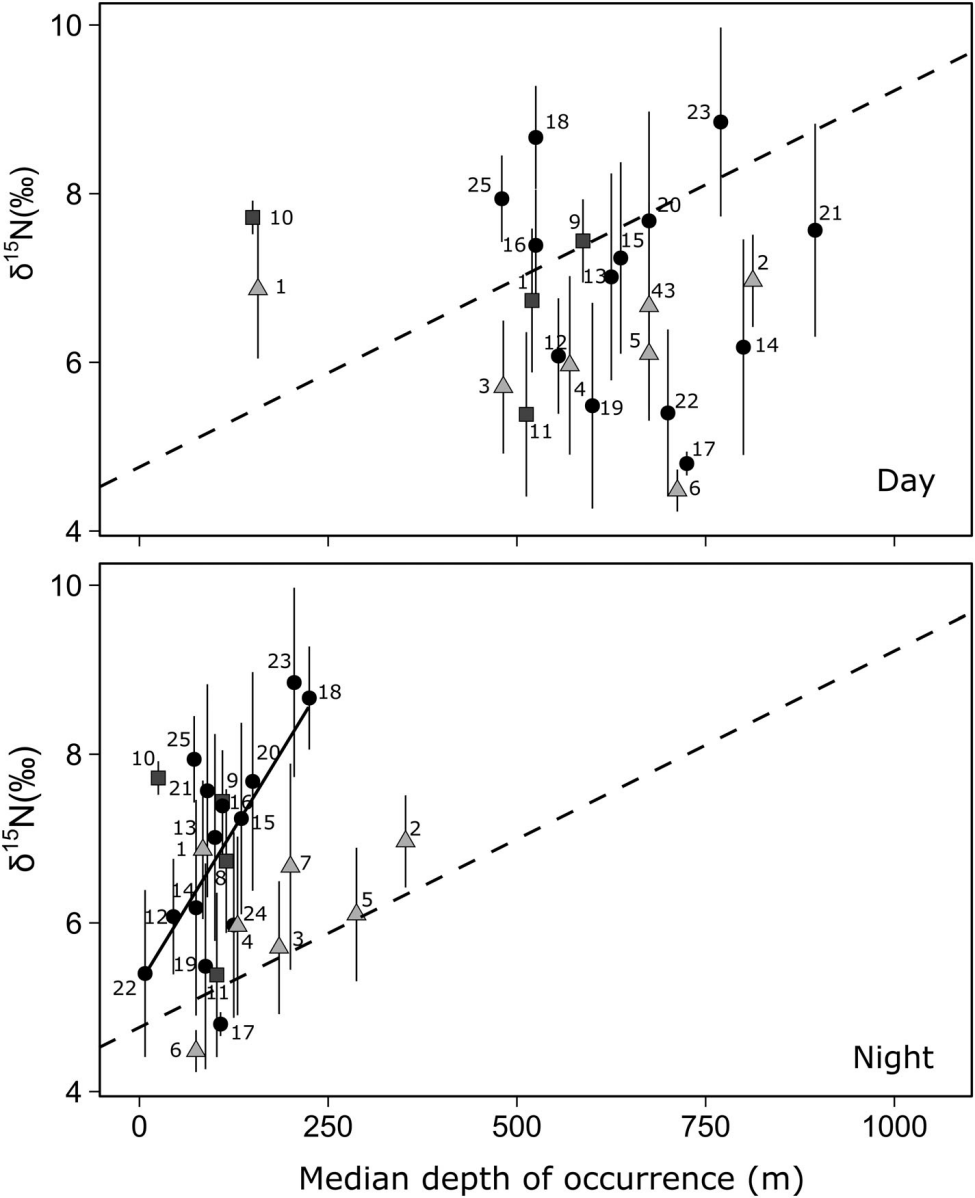
*δ* ^15^N values and median day and night depth of occurrence for vertically migrating species (black circles and black line, Actinopterygii; squares, Cephalopoda; and triangles, Malacostraca). The dashed line is the fitted regression of the relationship between *δ* ^15^N values and median depth of occurrence for nonmigrating species (as depicted from Fig. 1). Numbers correspond to species as in Table 1.

**Figure 3:**
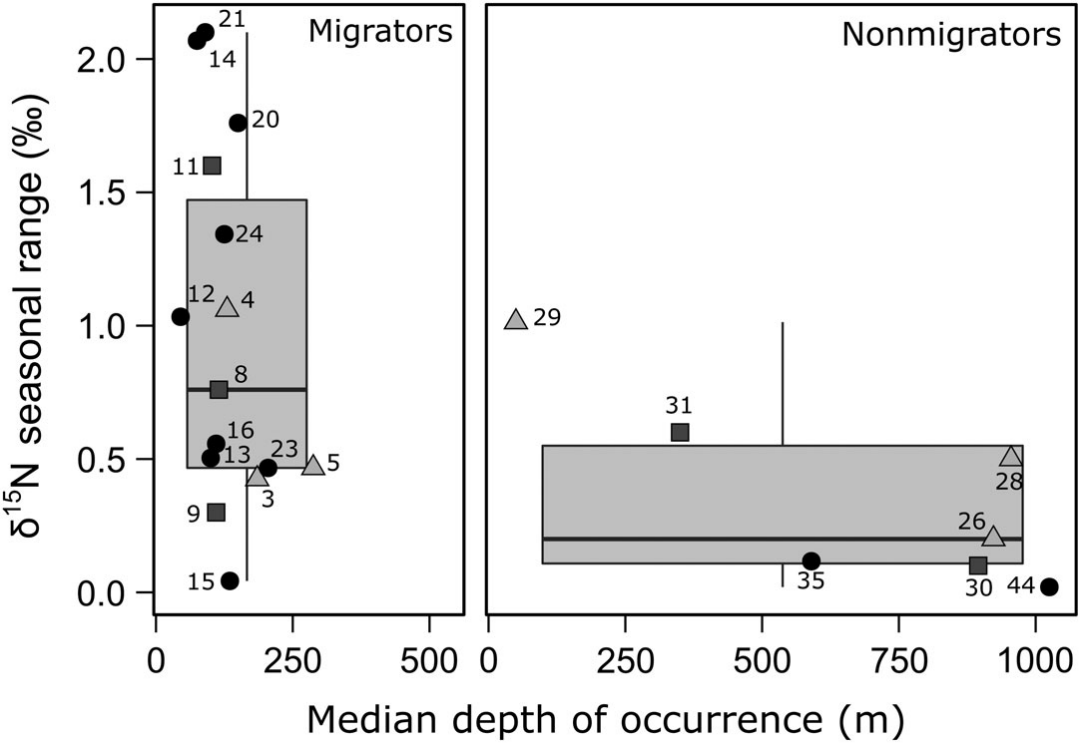
Range of variation between spring and summer in *δ* ^15^N values of migrating and nonmigrating micronekton species (black circles, Actinopterygii; squares, Cephalopoda; and triangles, Malacostraca). For each species, the depth of occurrence (night depth of occurrence for migrators) is represented. Numbers correspond to species as in Table 1.

In contrast, the *δ* ^15^N values of migratory species were not correlated to their depth of occurrence neither during the day nor at nighttime (Fig. 2) and were higher than the *δ* ^15^N values of shallow living nonmigrators (ANCOVA *p* < 0.05). When we included the taxonomic group (i.e., the Class of each species) as a factor in the *δ* ^15^N vs. night depth analysis of migrators, we found that the increase in *δ* ^15^N values was significant for fish (i.e., class Actinopterygii; *δ* ^15^N = 5.27 ± 0.56 + 0.015 ± 0.005 × nighttime depth, *R* ^2^ = 0.46, *p* < 0.001; Fig. 2), and the slope was significantly higher than that of all nonmigrators (ANCOVA *p* < 0.05). The difference in *δ* ^15^N values between samples collected in spring and summer was not significant (*t*‐test, *t* = −0.15; df = 45, *p* > 0.05); however, the average seasonal range of *δ* ^15^N values within species was significantly higher for migrators than for nonmigrators (*t*‐test, *t* = 3.6; df = 24, *p <* 0.001; Fig. 3). Also, for nonmigrators, the seasonal range of *δ* ^15^N values was negatively correlated with depth of occurrence (*δ* ^15^N seasonal range = 0.92 ± 0.16–0.00082 ± 0.00021 depth, *n* = 8, *R* ^2^ = 0.68, *p* < 0.001).

### Size‐based trophic structure

**Figure 4:**
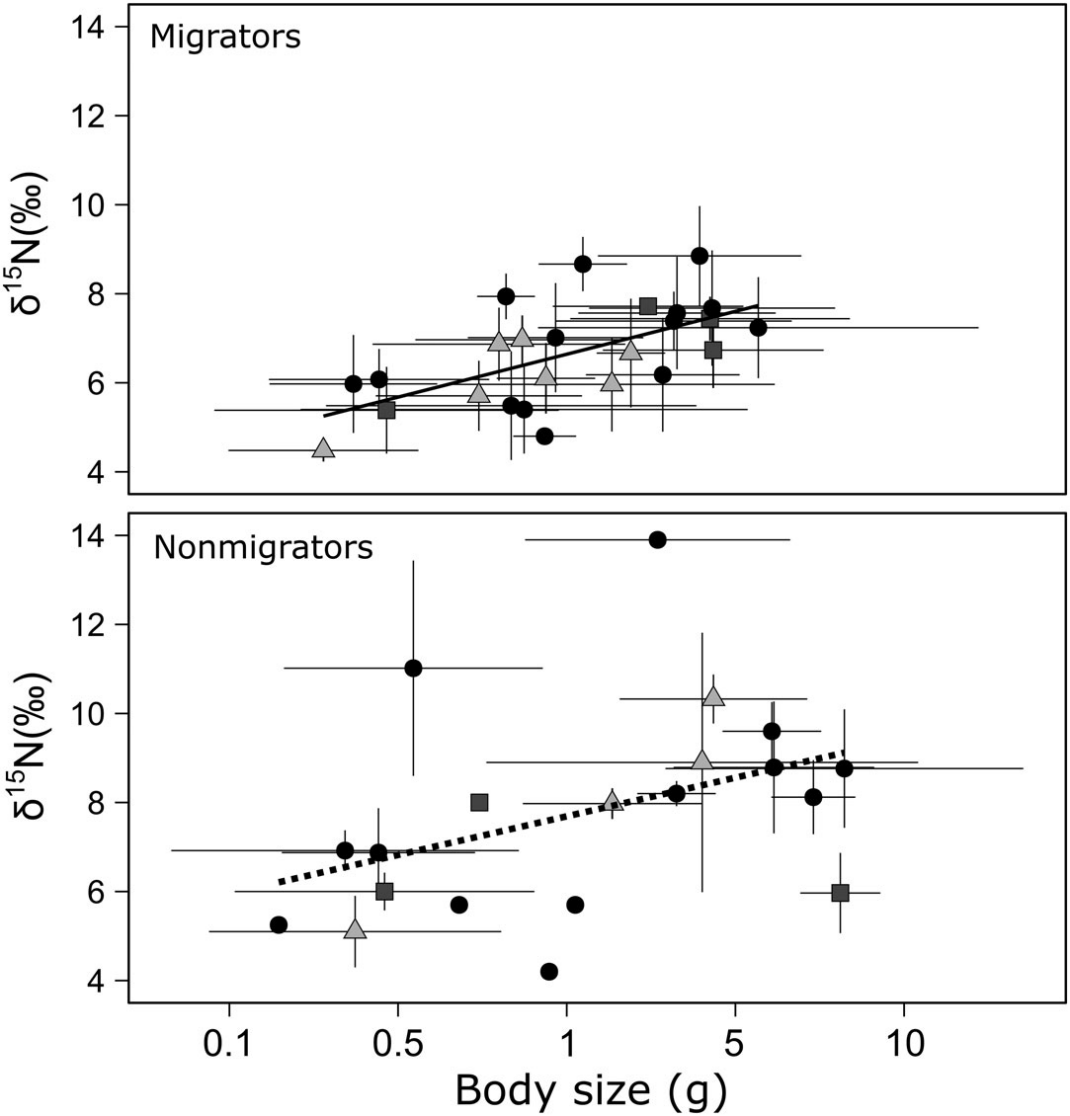
Relationship between *δ* ^15^N values and body size for migrating and nonmigrating micronekton species (black circles, Actinopterygii; squares, Cephalopoda; and triangles, Malacostraca). Dotted line represents a nonsignificant regression (*p* > 0.05).

**Figure 5:**
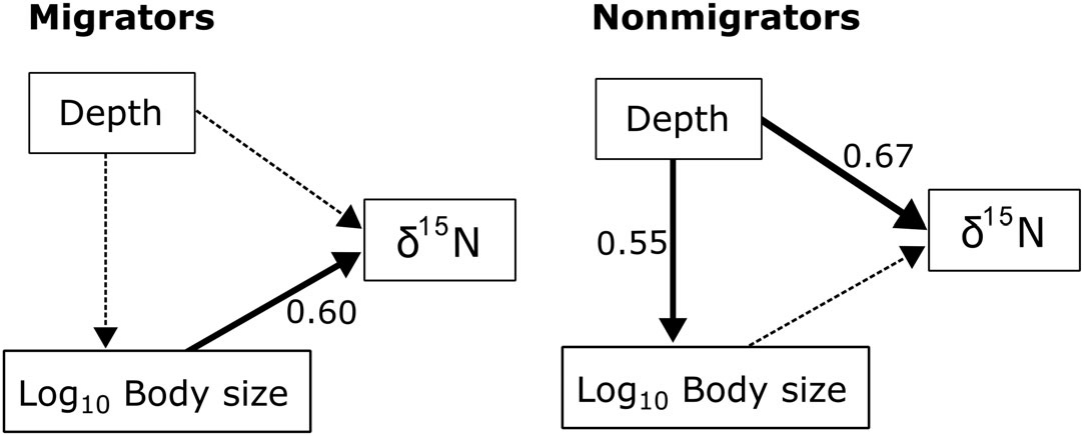
Path diagrams representing how depth of occurrence (night depth of occurrence for migrators) and body size influence the *δ* ^15^N values of migratory and nonmigratory species. Solid arrows represent significant relationships (*p* < 0.001) and dashed lines nonsignificant relationships. Values of standardized partial regression coefficients are shown.

We found that *δ* ^15^N values of migrators were largely explained by their body size (*δ* ^15^N = 6.64 ± 0.18 + 1.93 ± 0.51 log_10_ body size; *R* ^2^ = 0.38; *p* < 0.001; Fig. 4). Assuming a trophic fractionation of 3.4‰, the slope of the *δ* ^15^N vs. log_10_‐transformed body size relationship for migrating micronekton yielded a predator–prey mass ratio of 59 : 1 (PPMR = 10^(3.4/slope)^; Jennings et al. 2002). In the case of nonmigrators, *δ* ^15^N values were not significantly correlated to body size (*δ* ^15^N = 7.69 ± 0.49 + 1.74 ± 0.92 log_10_ body size; *R* ^2^ = 0.16; *p* > 0.07). The SEM analysis showed that only body size significantly explained variability in *δ* ^15^N values of all migrators, and depth did not have a direct or indirect effect on their *δ* ^15^N values (Fig. 5). The SEM analysis showed that although the nonmigrating micronekton species sampled were not size‐structured, deeper dwelling species were larger and had higher *δ* ^15^N values (Fig. 5).

## *Discussion*

We found clear evidence of differences in the food habits and dietary sources of migrating and nonmigrating micronekton, as reflected in their bulk stable nitrogen isotope compositions. Differences in the source of organic matter between some species of micronekton has been described previously (Valls et al. 2014; Choy et al. 2015; McClain‐Counts et al. 2017; Gloeckler et al. 2018), but this is the first time that a comprehensive dataset was used to resolve depth‐related, size‐based, and seasonal differences in the *δ* ^15^N values between species of micronekton driven by DVM. We hypothesized that nonmigrating species would show an increase in bulk tissue *δ* ^15^N values with depth, as has been previously described for particulate organic matter (Casciotti et al. 2008), zooplankton (Hannides et al. 2013) and benthic communities (Bergmann et al. 2009), whereas vertically migrating micronekton would exhibit similar *δ* ^15^N values to those of epipelagic nonmigrators. However, that was not true for migrators whose feeding depth was not constrained to surface waters, wherein their depth of occurrence does not resolve observed differences in bulk *δ* ^15^N values.

The *δ* ^15^N values of nonmigrating micronekton increased with median depth of occurrence with a pattern mirrored in zooplankton (Fig. 1), confirming zooplankton as the main prey resource of micronekton (Hopkins et al. 1997) and the main driver of the depth‐related increase in *δ* ^15^N values. For a given depth, micronekton was on average ∼0.77 trophic levels higher than zooplankton (assuming a trophic fractionation of 3.4‰; Fig. 1). We expect a slightly lower than one trophic level difference given that *δ* ^15^N values of zooplankton comprise individuals with a TP ranging from 2.1 to 3.1 (Hannides et al. 2013), and some micronekton species have a TP slightly lower than 3 (Choy et al. 2015; Gloeckler et al. 2018). The increase in *δ* ^15^N values with zooplankton depth has been well‐described in this (Hannides et al. 2013) and other ecosystems (Koppelmann et al. [2009], in the Mediterranean; Laakmann and Auel [2010], in the South Atlantic Ocean). This result emphasizes that identifying the relative influence of food web baseline processes is essential to understand the trophic ecology of higher trophic level micronekton, as those processes are reflected in their stable isotopic composition (Mintenbeck et al. 2007; Choy et al. 2012). Hannides et al. (2013) used AA‐CSIA, that allow changes in *δ* ^15^N values due to TP to be distinguished from changes at the base of the food web (Chikaraishi et al. 2009). These results demonstrated that the increase in *δ* ^15^N values of zooplankton with depth collected in the summertime at Sta. ALOHA was due to both a ^15^N enrichment at the base of the food web and a higher trophic level of deep‐water zooplankton.

DVM had a strong effect on zooplankton *δ* ^15^N values particularly in the upper mesopelagic zone (200–700 m; Fig. 1), where migrant biomass is greater (Angel et al. 1982; Hannides et al. 2013). The *δ* ^15^N values of zooplankton collected at night in that depth range largely reflect the nonmigrant portion of the community. Meanwhile during the day, the *δ* ^15^N values of zooplankton included migrators that had been feeding at the surface on organic matter with lower *δ* ^15^N values at night, and then descended to the mesopelagic yielding lower average *δ* ^15^N values at 200–700 m during the day. These distinct *δ* ^15^N values between migrant and nonmigrant zooplankton could be attributed to nonmigrants feeding on particles at depth that were enriched in ^15^N relative to surface particles, and their higher trophic level (Hannides et al. 2013). Overall, zooplankton *δ* ^15^N values clearly show differences in feeding depths between migrators and mesopelagic residents and supports their role in the active transport of organic matter produced in the surface layer to deeper waters through DVM.

Similar to zooplankton, we found that some migrating mesopelagic micronekton exhibited lower *δ* ^15^N values than those of mesopelagic nonmigrators (as in Valls et al. 2014; McClain‐Counts et al. 2017; Fig. 2). It is tempting to attribute the difference to the expected feeding of migrating species close to the surface on zooplankton with low *δ* ^15^N values. However, for most migrators, *δ* ^15^N values were higher than those of epipelagic nonmigrators (Fig. 2). This result could arise from: (i) a higher trophic level of migrating micronekton; (ii) differences in energetics between migrators and nonmigrators; or (iii) migrators feeding near the surface at night and also on prey found at greater depths during the day that had *δ* ^15^N values higher than epipelagic prey.

We evaluate each of these possibilities. First, obtaining TP from bulk *δ* ^15^N values would be largely biased by depth‐related changes in the isotopic composition of the baseline (Mintenbeck et al. 2007). However, Gloeckler et al. (2018) calculated TP for some of the species of micronekton included in this study using AA‐CSIA, so those estimates were not influenced by differences at the base of the food web. Migrators had a TP spanning a wide range from 2.6 to 4.5, and differences with epipelagic nonmigrators (median depth of occurrence = 0–200 m; TP = 2.8–3.5) were not significant (*t* = 0.87, df = 10.5, *p* > 0.1). TP does not appear to explain why the *δ* ^15^N values of migrators are higher than resident nonmigratory epipelagic species. Second, growth rate is positively correlated to the efficiency with which animals can utilize nitrogen (Trueman et al. 2005). Thus, higher growth rates imply a higher proportion of amino acids assimilated from dietary proteins, which would theoretically lead to *δ* ^15^N values that are more similar to those of diet and a lower trophic fractionation (Trueman et al. 2005; Caut et al. 2009). Migratory fishes have a higher feeding rate and growth rate than nonmigratory species (Childress et al. 1980; Koslow 1996; Moku et al. 2000). Hence, contrary to our results, migrators would be expected to have *δ* ^15^N values lower than those of nonmigrators feeding on a similar diet. Therefore energy needs and inherent differences in the trophic isotope discrimination factor are likely not responsible for the consistently higher *δ* ^15^N values found for migrators relative to epipelagic nonmigrators.

Accordingly, our results strongly suggest that the diet of vertical migrators includes both epipelagic or vertically migrating zooplankton captured at night, as well as nonmigrating zooplankton consumed at depth during the daytime. We used median depth of occurrence found in the literature for each species to explore differences in the stable isotope composition, because the depth of capture of the particular few individuals used in the analysis might not represent an actual population distribution pattern. There is also individual variability in DVM (Hays et al. 2001; Pearre 2003), which leads to asynchronous migrations within populations (Pearre 1979). As a result, there can be individuals of vertically migrating species not performing DVM usually due to their greater age or size (Kaartvedt et al. 2009), which could lead to intra‐specific variations in *δ* ^15^N values. However, we analyzed individuals within a narrow body size range, so differences in the migrating behavior due to size are unlikely. Despite being often ignored, previous work has reported vertical migrators feeding partially at their daytime depth (e.g., Tyler and Pearcy 1975; Moku et al. 2000; Clarke and Tyler 2008). This behavior could result in some upward transport of organic matter, leading to a recycling of nutrients in the euphotic zone. This could have a significant effect on carbon export flux that should be considered in future investigations of carbon budgets.

Contrary to what was expected, among migratory micronekton, only fishes (class Actinopterygii) showed an increase in *δ* ^15^N values with night depth of occurrence (Fig. 2), which points to taxonomic group as an important factor determining food habits for vertical migrating micronekton. Moreover, the high slope of the *δ* ^15^N values vs. night depth relationship of migratory fish suggests that the proportion of total feeding performed at night by migrating fishes depend markedly on the depth range within the epipelagic zone reached during the night. That is, fishes that ascend closer to the surface at night have a lower dependence on organic matter at depth, which is ^15^N‐enriched relative to that at the surface, suggesting that they feed at night to a higher extent than fish that migrate only to a deeper depth range of the epipelagic zone. This can be due to the exponential decline in zooplankton biomass with depth (Hannides et al. 2013) so that only those fishes migrating closer to the surface are able to meet their energy needs feeding exclusively during the night.

A large variance in migrator *δ* ^15^N values was largely explained by body size (Fig. 4), as opposed to depth, indicating that micronekton performing DVM feed on prey that mostly depend on the same source of organic matter (Layman et al. 2005). The PPMR obtained from the slope of the *δ* ^15^N values vs. body size relationship for migratory micronekton (59 : 1) was lower than previous estimates for a fish community (424 : 1; Jennings and Mackinson 2003). The PPMR is expected to increase with predator size (Barnes et al. 2010) and also vertically migrating predators could be selecting large prey, probably as a response to the higher energetic requirements. The selectivity in prey size together with the low PPMR are characteristic of longer food webs (Jennings and Warr 2003), which is in agreement with the wider range of TPs described for migrating micronekton as compared to nonmigrating species (Gloeckler et al. 2018).

Conversely, nonmigrators were not significantly size structured (Figs. 4, 5) although deeper dwelling species were larger (Fig. 5). Body size and depth can have confounding effects that should be carefully considered in size‐based studies of trophic structure encompassing a wide depth range, or including species with “bigger–deeper” patterns (Collins et al. 2005). Nonmigrators may encounter a lower prey availability that leads to more generalist diets of some species, or to diets based on different sources of organic matter. Moreover, the variance in *δ* ^15^N values increased with depth of occurrence of nonmigrators (Fig. 1), so those different feeding habits were more marked at greater depths. This matches the described dependence of some bathypelagic micronekton on a very small/suspended microbially altered particle based food web, in contrast to epipelagic species that depend on organic matter produced by fresh phytoplankton (Choy et al. 2015; Gloeckler et al. 2018). Very small and/or suspended particles have high *δ* ^15^N values, which will be incorporated in the zooplankton that feed on them (Mintenbeck et al. 2007), ultimately leading to positive residuals for the linear relationship between the *δ* ^15^N values vs. depth of occurrence for micronekton, like in the mesopelagic fish *Cyclothone pallida* or the bathypelagic fish *Melanocetus johnsonii*.

Despite significant seasonal changes in surface particle *δ* ^15^N values (Dore et al. 2002) and zooplankton biomass and isotopic values (Bode and Alvarez‐Ossorio 2004; Valencia et al. 2016), we did not find a significant difference in *δ* ^15^N values of micronekton between spring and summer (see also Gloeckler et al. 2018), probably due to their tissue turnover rates integrating feeding across several months or more (Vander Zanden et al. 2015). While mean values did not vary seasonally, *δ* ^15^N values of most migrating species did show a higher range in *δ* ^15^N values than nonmigrators (Fig. 3), which makes sense because migrators have a higher metabolic rate (Drazen and Seibel 2007) so they are expected to reflect *δ* ^15^N values of a new diet quicker than fishes with a lower metabolic rate (Trueman et al. 2005). We also found that the range of variation in *δ* ^15^N values of nonmigrators was depth‐dependent, with epipelagic species showing a higher difference between spring and summer. The central North Pacific Ocean seasonal cycle is characterized by a summer increase in primary production (Church et al. 2013) coupled with an export pulse that transports carbon to the deep sea (Karl et al. 2012). As a result, the mesopelagic and bathypelagic systems in the study area also exhibit seasonal variations that are reflected in the biomass and stable isotope composition of zooplankton (C. C. S. Hannides et al. unpubl.). This suggests that depth‐related changes in the seasonal variations of the range of *δ* ^15^N values of nonmigrators were due to the decline with depth of their metabolic rate (Drazen and Seibel 2007) rather than to the environmental conditions.

In summary, we found marked differences in the food habits and dietary sources of micronekton species driven by vertical migration. Variations in the isotopic composition of nonmigrating micronekton were largely explained by its depth of occurrence, a pattern mirrored in zooplankton. Also, the higher variance in *δ* ^15^N values with depth points to the increasing reliance of deeper dwelling nonmigrating micronekton on microbially reworked suspended particles (Hannides et al. 2013; Gloeckler et al. 2018). In contrast, migrators had a more selective diet of large prey and exhibited a higher range of variation in *δ* ^15^N values seasonally than nonmigrators probably because they are expected to have higher metabolic rates and thus higher tissue turnover rates. Migrators also had *δ* ^15^N values intermediate between epipelagic and lower mesopelagic nonmigrators, which indicates that migrators feed in deeper water during the day and not only at night when they ascend closer to the surface. The importance of daytime feeding in the diet of migratory fish was higher for species migrating into the lower epipelagic zone than for those migrating up to the surface during the night, probably due to the lower prey availability.

## Conflict of Interest:

None declared

## Supporting information


**Appendix S1**: Supplementary Information.
