# Supplementary material for: Differences in the trophic ecology of micronekton driven by diel vertical migration
Source: Limnol Oceanogr. 2019 Feb 4;64(4):1473–83. doi: 10.1002/lno.11128 (PMC6774321; doi:10.1002/lno.11128)
Supplement: Supplementary file 1 — Appendix S1: Supplementary Information. [file LNO-64-1473-s001.docx]

**Supplementary material**

Figure S1. Map of the Hawaiian Islands showing the sampling stations (St. ALOHA, west O’ahu and Cross Seamount) of micronekton and zooplankton for this study.


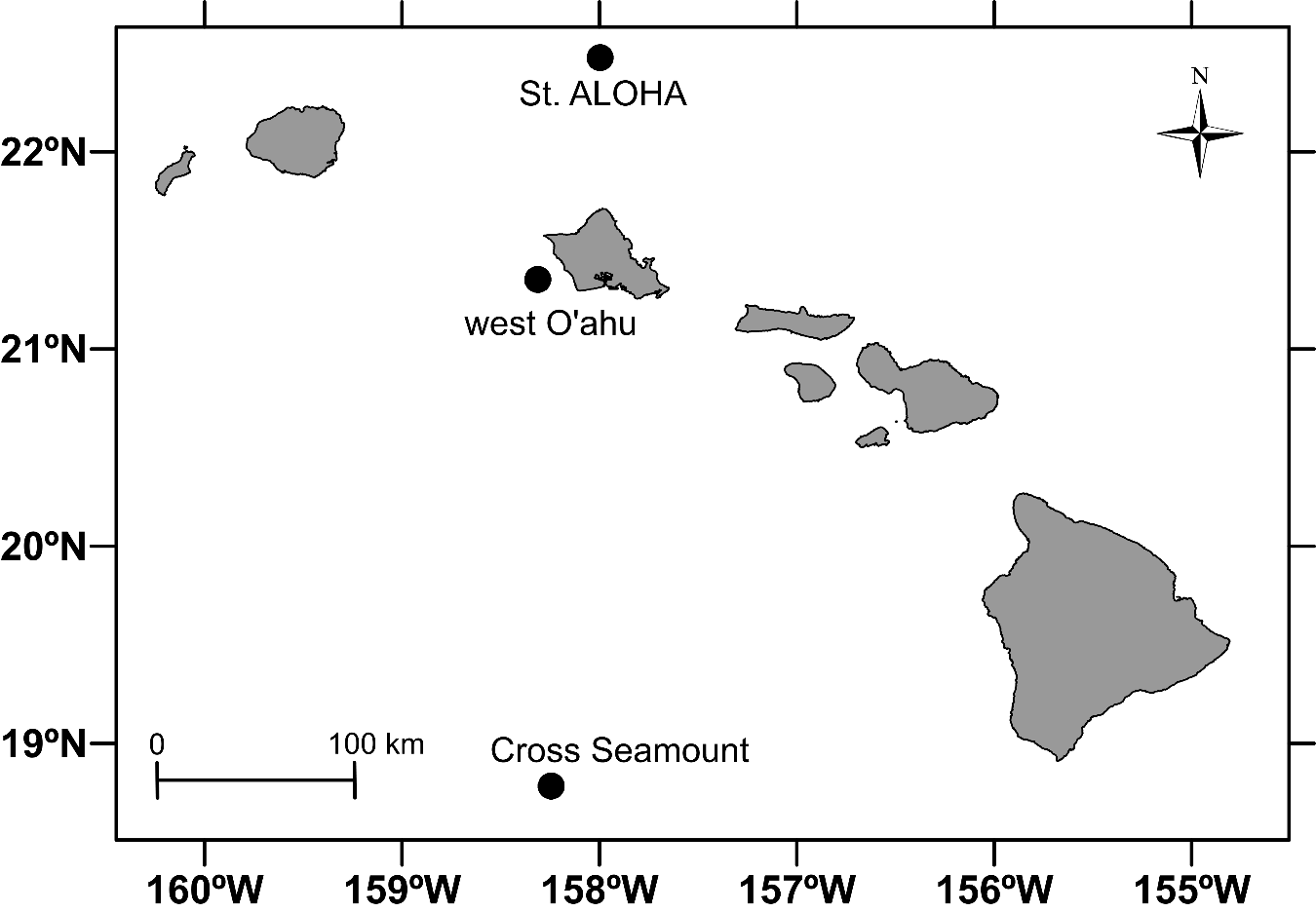


Table S1. Year, Month (M), Season (S: summer, Sp: spring, W: winter), Location, minimum and maximum sampling depth range, Gear and Night/Day information for each individual analyzed in this study.

| **Species** | **Year** | **M** | **Season** | | **Location** | **Min Depth** | **Max Depth** | **Gear** | **Night/Day** |
| --- | --- | --- | --- | --- | --- | --- | --- | --- | --- |
| *Abralia trigonura* | 2011 | 8 | S | W. Oahu | | 0 | 999 | MOCNESS | D |
| *Abralia trigonura* | 2008 | 4 | Sp | Cross Seamount | | 0 | 503 | Cobb Trawl | D |
| *Abralia trigonura* | 2008 | 4 | Sp | Cross Seamount | | 0 | 503 | Cobb Trawl | D |
| *Abralia trigonura* | 2008 | 4 | Sp | Cross Seamount | | 0 | 503 | Cobb Trawl | D |
| *Abralia trigonura* | 2008 | 4 | Sp | Cross Seamount | | 0 | 503 | Cobb Trawl | D |
| *Abralia trigonura* | 2007 | 4 | Sp | Cross Seamount | | 0 | 133 | Cobb Trawl | N |
| *Abraliopsis sp. A* | 2008 | 5 | Sp | Cross Seamount | | 0 | 202 | Cobb Trawl | N |
| *Abraliopsis sp. A* | 2007 | 4 | Sp | Cross Seamount | | 0 | 133 | Cobb Trawl | N |
| *Abraliopsis sp. A* | 2007 | 5 | Sp | Cross Seamount | | 0 | 184 | Cobb Trawl | N |
| *Abraliopsis sp. A* | 2007 | 5 | Sp | Cross Seamount | | 0 | 184 | Cobb Trawl | N |
| *Abraliopsis sp. A* | 2008 | 5 | Sp | Cross Seamount | | 0 | 202 | Cobb Trawl | N |
| *Acanthephyra curtirostris* | 2011 | 8 | S | St. ALOHA | | 1000 | 1500 | MOCNESS | N |
| *Acanthephyra curtirostris* | 2011 | 8 | S | W. Oahu | | 799 | 903 | MOCNESS | D |
| *Acanthephyra curtirostris* | 2011 | 8 | S | St. ALOHA | | 498 | 1000 | MOCNESS | N |
| *Acanthephyra curtirostris* | 2011 | 3 | Sp | W. Oahu | | 0 | 800 | Tucker | D |
| *Acanthephyra curtirostris* | 2011 | 8 | S | St. ALOHA | | 1000 | 1501 | MOCNESS | D |
| *Acanthephyra curtirostris* | 2011 | 3 | Sp | W. Oahu | | 0 | 800 | Tucker | D |
| *Acanthephyra curtirostris* | 2011 | 3 | Sp | W. Oahu | | 0 | 800 | Tucker | D |
| *Acanthephyra curtirostris* | 2011 | 8 | S | W. Oahu | | 701 | 799 | MOCNESS | D |
| *Argyropelecus hemigymnus* | 2011 | 8 | S | St. ALOHA | | 250 | 499 | MOCNESS | D |
| *Argyropelecus hemigymnus* | 2011 | 8 | S | St. ALOHA | | 250 | 499 | MOCNESS | D |
| *Argyropelecus hemigymnus* | 2011 | 8 | S | W. Oahu | | 500 | 599 | MOCNESS | D |
| *Argyropelecus hemigymnus* | 2011 | 8 | S | W. Oahu | | 248 | 498 | MOCNESS | N |
| *Argyropelecus sladeni* | 2011 | 3 | Sp | W. Oahu | | 0 | 800 | Tucker | D |
| *Benthosema suborbitale* | 2011 | 3 | Sp | W. Oahu | | 0 | 800 | Tucker | D |
| *Benthosema suborbitale* | 2011 | 8 | S | W. Oahu | | 500 | 599 | MOCNESS | D |
| *Benthosema suborbitale* | 2011 | 8 | S | W. Oahu | | 500 | 599 | MOCNESS | D |
| *Benthosema suborbitale* | 2011 | 8 | S | W. Oahu | | 2 | 248 | MOCNESS | N |
| *Bolinichthys distofax* | 2011 | 3 | Sp | W. Oahu | | 0 | 700 | Tucker | D |
| *Bolinichthys distofax* | 2011 | 8 | S | W. Oahu | | 599 | 701 | MOCNESS | D |
| *Bolinichthys distofax* | 2007 | 4 | Sp | Cross Seamount | | 0 | 626 | Cobb Trawl | D |
| *Bolinichthys distofax* | 2007 | 4 | Sp | Cross Seamount | | 0 | 626 | Cobb Trawl | D |
| *Bolinichthys distofax* | 2007 | 4 | Sp | Cross Seamount | | 0 | 626 | Cobb Trawl | D |
| *Bolinichthys distofax* | 2007 | 4 | Sp | Cross Seamount | | 0 | 626 | Cobb Trawl | D |
| *Bolinichthys distofax* | 2007 | 4 | Sp | Cross Seamount | | 0 | 626 | Cobb Trawl | D |
| *Bolinichthys distofax* | 2011 | 8 | S | W. Oahu | | 500 | 599 | MOCNESS | D |
| *Bolinichthys longipes* | 2011 | 8 | S | W. Oahu | | 500 | 599 | MOCNESS | D |
| *Bolinichthys longipes* | 2011 | 3 | Sp | W. Oahu | | 0 | 200 | Tucker | N |
| *Bolinichthys longipes* | 2011 | 3 | Sp | W. Oahu | | 0 | 700 | Tucker | D |
| *Bolinichthys longipes* | 2011 | 3 | Sp | W. Oahu | | 0 | 200 | Tucker | N |
| *Bolinichthys longipes* | 2011 | 3 | Sp | W. Oahu | | 0 | 800 | Tucker | D |
| *Bolinichthys longipes* | 2010 | 4 | Sp | W. Oahu | | 0 | 250 | IKMT | N |
| *Bolinichthys longipes* | 2008 | 4 | Sp | Cross Seamount | | 0 | 155 | Cobb Trawl | N |
| *Bolinichthys longipes* | 2011 | 3 | Sp | W. Oahu | | 0 | 800 | Tucker | D |
| *Bolinichthys longipes* | 2011 | 3 | Sp | W. Oahu | | 0 | 200 | Tucker | N |
| *Bolinichthys longipes* | 2011 | 3 | Sp | W. Oahu | | 0 | 800 | Tucker | D |
| *Bolinichthys longipes* | 2008 | 4 | Sp | Cross Seamount | | 0 | 155 | Cobb Trawl | N |
| *Bolinichthys longipes* | 2008 | 4 | Sp | Cross Seamount | | 0 | 155 | Cobb Trawl | N |
| *Bolinichthys longipes* | 2008 | 4 | Sp | Cross Seamount | | 0 | 155 | Cobb Trawl | N |
| *Bolinichthys longipes* | 2011 | 8 | S | W. Oahu | | 2 | 248 | MOCNESS | N |
| *Bolinichthys longipes* | 2008 | 4 | Sp | Cross Seamount | | 0 | 155 | Cobb Trawl | N |
| *Ceratoscopelus warmingii* | 2011 | 8 | S | St. ALOHA | | 2 | 247 | MOCNESS | N |
| *Ceratoscopelus warmingii* | 2011 | 8 | S | St. ALOHA | | 2 | 247 | MOCNESS | N |
| *Ceratoscopelus warmingii* | 2011 | 8 | S | St. ALOHA | | 2 | 247 | MOCNESS | N |
| *Ceratoscopelus warmingii* | 2011 | 3 | Sp | W. Oahu | | 0 | 200 | Tucker | N |
| *Ceratoscopelus warmingii* | 2011 | 3 | Sp | W. Oahu | | 0 | 1500 | Tucker | N |
| *Ceratoscopelus warmingii* | 2011 | 3 | Sp | W. Oahu | | 0 | 250 | Tucker | N |
| *Ceratoscopelus warmingii* | 2010 | 5 | Sp | W. Oahu | | 0 | 700 | IKMT | N |
| *Ceratoscopelus warmingii* | 2007 | 4 | Sp | Cross Seamount | | 0 | 133 | Cobb Trawl | N |
| *Ceratoscopelus warmingii* | 2007 | 4 | Sp | Cross Seamount | | 0 | 133 | Cobb Trawl | N |
| *Ceratoscopelus warmingii* | 2007 | 4 | Sp | Cross Seamount | | 0 | 133 | Cobb Trawl | N |
| *Ceratoscopelus warmingii* | 2007 | 4 | Sp | Cross Seamount | | 0 | 133 | Cobb Trawl | N |
| *Ceratoscopelus warmingii* | 2007 | 4 | Sp | Cross Seamount | | 0 | 133 | Cobb Trawl | N |
| *Ceratoscopelus warmingii* | 2007 | 4 | Sp | Cross Seamount | | 0 | 133 | Cobb Trawl | N |
| *Ceratoscopelus warmingii* | 2010 | 4 | Sp | W. Oahu | | 0 | 250 | IKMT | N |
| *Ceratoscopelus warmingii* | 2007 | 4 | Sp | Cross Seamount | | 0 | 133 | Cobb Trawl | N |
| *Ceratoscopelus warmingii* | 2007 | 4 | Sp | Cross Seamount | | 0 | 133 | Cobb Trawl | N |
| *Ceratoscopelus warmingii* | 2007 | 4 | Sp | Cross Seamount | | 0 | 626 | Cobb Trawl | D |
| *Ceratoscopelus warmingii* | 2007 | 4 | Sp | Cross Seamount | | 0 | 133 | Cobb Trawl | N |
| *Ceratoscopelus warmingii* | 2011 | 3 | Sp | W. Oahu | | 0 | 800 | Tucker | D |
| *Ceratoscopelus warmingii* | 2010 | 4 | Sp | W. Oahu | | 0 | 250 | IKMT | N |
| *Chauliodus sloani* | 2011 | 8 | S | St. ALOHA | | 398 | 596 | MOCNESS | D |
| *Chauliodus sloani* | 2011 | 8 | S | W. Oahu | | 599 | 701 | MOCNESS | D |
| *Chauliodus sloani* | 2011 | 8 | S | W. Oahu | | 599 | 701 | MOCNESS | D |
| *Chauliodus sloani* | 2011 | 3 | Sp | W. Oahu | | 0 | 800 | Tucker | D |
| *Chauliodus sloani* | 2011 | 8 | S | W. Oahu | | 599 | 701 | MOCNESS | D |
| *Chauliodus sloani* | 2011 | 8 | S | St. ALOHA | | 250 | 499 | MOCNESS | D |
| *Chauliodus sloani* | 2014 | 8 | S | St. ALOHA | | 0 | 1500 | MOCNESS | N |
| *Chauliodus sloani* | 2014 | 8 | S | St. ALOHA | | 500 | 700 | MOCNESS | D |
| *Cyclothone alba* | 2011 | 8 | S | St. ALOHA | | 2001 | 2502 | MOCNESS | N |
| *Cyclothone alba* | 2011 | 8 | S | St. ALOHA | | 398 | 596 | MOCNESS | D |
| *Cyclothone alba* | 2011 | 8 | S | St. ALOHA | | 398 | 596 | MOCNESS | D |
| *Cyclothone alba* | 2014 | 8 | S | St. ALOHA | | 500 | 700 | MOCNESS | D |
| *Cyclothone alba* | 2014 | 8 | S | St. ALOHA | | 500 | 700 | MOCNESS | N |
| *Cyclothone pallida* | 2011 | 8 | S | St. ALOHA | | 596 | 796 | MOCNESS | D |
| *Cyclothone pallida* | 2011 | 8 | S | St. ALOHA | | 596 | 796 | MOCNESS | D |
| *Cyclothone pallida* | 2011 | 8 | S | St. ALOHA | | 596 | 796 | MOCNESS | D |
| *Cyclothone pallida* | 2011 | 8 | S | W. Oahu | | 998 | 1251 | MOCNESS | N |
| *Cyclothone pallida* | 2011 | 8 | S | St. ALOHA | | 596 | 796 | MOCNESS | D |
| *Cyclothone pallida* | 2011 | 8 | S | W. Oahu | | 998 | 1251 | MOCNESS | N |
| *Cyclothone pallida* | 2011 | 8 | S | W. Oahu | | 998 | 1251 | MOCNESS | N |
| *Cyclothone pallida* | 2014 | 8 | S | St. ALOHA | | 500 | 700 | MOCNESS | D |
| *Cyclothone pallida* | 2014 | 8 | S | St. ALOHA | | 500 | 700 | MOCNESS | N |
| *Cyclothone pallida* | 2014 | 8 | S | St. ALOHA | | 1000 | 1500 | MOCNESS | N |
| *Cyclothone pallida* | 2014 | 2 | W | St. ALOHA | | 500 | 700 | MOCNESS |  |
| *Cyclothone pallida* | 2014 | 2 | W | St. ALOHA | | 500 | 700 | MOCNESS |  |
| *Cyclothone pallida* | 2014 | 8 | S | St. ALOHA | | 1000 | 1500 | MOCNESS | D |
| *Cyclothone pallida* | 2014 | 8 | S | St. ALOHA | | 1000 | 1500 | MOCNESS | N |
| *Cyclothone pallida* | 2014 | 8 | S | St. ALOHA | | 700 | 1000 | MOCNESS | N |
| *Cyclothone pallida* | 2014 | 8 | S | St. ALOHA | | 1000 | 1500 | MOCNESS | N |
| *Cyclothone pallida* | 2014 | 8 | S | St. ALOHA | | 700 | 1000 | MOCNESS | N |
| *Cyclothone pallida* | 2014 | 8 | S | St. ALOHA | | 700 | 1000 | MOCNESS | N |
| *Cyema atrum* | 2011 | 8 | S | St. ALOHA | | 0 | 0 | MOCNESS | D |
| *Cyema atrum* | 2011 | 8 | S | St. ALOHA | | 1000 | 1502 | MOCNESS | N |
| *Cyema atrum* | 2011 | 8 | S | St. ALOHA | | 1000 | 1501 | MOCNESS | D |
| *Diaphus perspicillatus* | 2011 | 3 | Sp | W. Oahu | | 0 | 200 | Tucker | N |
| *Diaphus perspicillatus* | 2010 | 5 | Sp | W. Oahu | | 0 | 950 | IKMT | D |
| *Diaphus perspicillatus* | 2007 | 4 | Sp | Cross Seamount | | 0 | 133 | Cobb Trawl | N |
| *Diaphus perspicillatus* | 2007 | 4 | Sp | Cross Seamount | | 0 | 133 | Cobb Trawl | N |
| *Diaphus perspicillatus* | 2007 | 4 | Sp | Cross Seamount | | 0 | 133 | Cobb Trawl | N |
| *Diaphus perspicillatus* | 2007 | 4 | Sp | Cross Seamount | | 0 | 133 | Cobb Trawl | N |
| *Diaphus perspicillatus* | 2007 | 4 | Sp | Cross Seamount | | 0 | 133 | Cobb Trawl | N |
| *Diaphus perspicillatus* | 2007 | 4 | Sp | Cross Seamount | | 0 | 133 | Cobb Trawl | N |
| Euphausiidae | 2014 | 2 | W | St. ALOHA | | 0 | 1500 | MOCNESS | N |
| Euphausiidae | 2014 | 2 | W | St. ALOHA | | 500 | 700 | MOCNESS | D |
| Euphausiidae | 2014 | 2 | W | St. ALOHA | | 100 | 500 | MOCNESS | D |
| Euphausiidae | 2007 | 4 | Sp | Cross Seamount | | 0 | 133 | Cobb Trawl | N |
| Euphausiidae | 2007 | 4 | Sp | Cross Seamount | | 0 | 133 | Cobb Trawl | N |
| Euphausiidae | 2011 | 3 | Sp | W. Oahu | | 0 | 1000 | Tucker | N |
| *Eurypharynx pelecanoides* | 2011 | 8 | S | St. ALOHA | | 1000 | 1502 | MOCNESS | N |
| *Eurypharynx pelecanoides* | 2011 | 8 | S | St. ALOHA | | 1000 | 1502 | MOCNESS | N |
| *Eustomias bifilis* | 2011 | 8 | S | St. ALOHA | | 498 | 1000 | MOCNESS | N |
| *Eustomias bifilis* | 2011 | 8 | S | St. ALOHA | | 1000 | 1500 | MOCNESS | N |
| *Exocoetus volitans* | 2011 | 8 | S | St. ALOHA | | 1 | 2502 | MOCNESS | N |
| *Gempylus serpens* | 2008 | 5 | Sp | Cross Seamount | | 0 | 202 | Cobb Trawl | N |
| *Gempylus serpens* | 2008 | 5 | Sp | Cross Seamount | | 0 | 202 | Cobb Trawl | N |
| *Gennadas bouvieri* | 2011 | 8 | S | W. Oahu | | 701 | 799 | MOCNESS | D |
| *Gennadas bouvieri* | 2011 | 8 | S | St. ALOHA | | 248 | 501 | MOCNESS | N |
| *Gennadas bouvieri* | 2011 | 8 | S | W. Oahu | | 701 | 799 | MOCNESS | D |
| *Gennadas bouvieri* | 2011 | 8 | S | St. ALOHA | | 796 | 999 | MOCNESS | D |
| *Gennadas bouvieri* | 2011 | 8 | S | W. Oahu | | 248 | 498 | MOCNESS | N |
| *Gennadas bouvieri* | 2011 | 8 | S | St. ALOHA | | 250 | 499 | MOCNESS | D |
| *Gnathophausia ingens* | 2011 | 3 | Sp | W. Oahu | | 0 | 800 | Tucker | D |
| *Gnathophausia ingens* | 2011 | 3 | Sp | W. Oahu | | 0 | 700 | Tucker | D |
| *Gnathophausia ingens* | 2011 | 3 | Sp | W. Oahu | | 0 | 1500 | Tucker | N |
| *Gonostoma atlanticum* | 2011 | 8 | S | W. Oahu | | 2 | 248 | MOCNESS | N |
| *Gonostoma atlanticum* | 2011 | 8 | S | W. Oahu | | 2 | 248 | MOCNESS | N |
| *Gonostoma atlanticum* | 2011 | 8 | S | W. Oahu | | 500 | 599 | MOCNESS | D |
| *Hyaloteuthis pelagica* | 2007 | 5 | Sp | Cross Seamount | | 0 | 184 | Cobb Trawl | N |
| *Hyaloteuthis pelagica* | 2007 | 5 | Sp | Cross Seamount | | 0 | 184 | Cobb Trawl | N |
| *Hyaloteuthis pelagica* | 2007 | 5 | Sp | Cross Seamount | | 0 | 184 | Cobb Trawl | N |
| *Hyaloteuthis pelagica* | 2007 | 5 | Sp | Cross Seamount | | 0 | 197 | Cobb Trawl | N |
| *Hyaloteuthis pelagica* | 2007 | 5 | Sp | Cross Seamount | | 0 | 184 | Cobb Trawl | N |
| *Hyaloteuthis pelagica* | 2007 | 5 | Sp | Cross Seamount | | 0 | 184 | Cobb Trawl | N |
| *Hyaloteuthis pelagica* | 2007 | 5 | Sp | Cross Seamount | | 0 | 197 | Cobb Trawl | N |
| *Hyaloteuthis pelagica* | 2007 | 5 | Sp | Cross Seamount | | 0 | 197 | Cobb Trawl | N |
| *Hyaloteuthis pelagica* | 2007 | 5 | Sp | Cross Seamount | | 0 | 197 | Cobb Trawl | N |
| *Hyaloteuthis pelagica* | 2007 | 5 | Sp | Cross Seamount | | 0 | 197 | Cobb Trawl | N |
| *Hyaloteuthis pelagica* | 2007 | 5 | Sp | Cross Seamount | | 0 | 197 | Cobb Trawl | N |
| *Hygophum proximum* | 2011 | 8 | S | St. ALOHA | | 2 | 247 | MOCNESS | N |
| *Hygophum proximum* | 2011 | 8 | S | St. ALOHA | | 2 | 247 | MOCNESS | N |
| *Hygophum proximum* | 2011 | 8 | S | W. Oahu | | 599 | 701 | MOCNESS | D |
| *Hygophum proximum* | 2011 | 8 | S | St. ALOHA | | 1 | 2502 | MOCNESS | N |
| *Hygophum proximum* | 2011 | 8 | S | W. Oahu | | 1 | 1251 | MOCNESS | N |
| *Hygophum proximum* | 2014 | 8 | S | St. ALOHA | | 700 | 1000 | MOCNESS | D |
| *Hygophum proximum* | 2014 | 8 | S | St. ALOHA | | 1000 | 1500 | MOCNESS | D |
| *Idiacanthus fasciola* | 2011 | 3 | Sp | W. Oahu | | 0 | 250 | Tucker | N |
| *Idiacanthus fasciola* | 2011 | 3 | Sp | W. Oahu | | 0 | 200 | Tucker | N |
| *Idiacanthus fasciola* | 2011 | 3 | Sp | W. Oahu | | 0 | 800 | Tucker | D |
| *Idiacanthus fasciola* | 2011 | 3 | Sp | W. Oahu | | 0 | 800 | Tucker | N |
| *Idiacanthus fasciola* | 2010 | 5 | Sp | W. Oahu | | 0 | 250 | IKMT | N |
| *Idiacanthus fasciola* | 2008 | 5 | Sp | Cross Seamount | | 0 | 202 | Cobb Trawl | N |
| *Idiacanthus fasciola* | 2008 | 5 | Sp | Cross Seamount | | 0 | 202 | Cobb Trawl | N |
| *Idiacanthus fasciola* | 2010 | 5 | Sp | NWHI | | 0 | 900 | Ring Net | D |
| *Idiacanthus fasciola* | 2008 | 5 | Sp | Cross Seamount | | 0 | 202 | Cobb Trawl | N |
| *Janicella spinicauda* | 2011 | 8 | S | St. ALOHA | | 9 | 250 | MOCNESS | D |
| *Janicella spinicauda* | 2011 | 3 | Sp | W. Oahu | | 0 | 800 | Tucker | D |
| *Janicella spinicauda* | 2011 | 3 | Sp | W. Oahu | | 0 | 800 | Tucker | D |
| *Janicella spinicauda* | 2011 | 3 | Sp |  | | 0 | 800 | Tucker | N |
| *Janicella spinicauda* | 2011 | 8 | S | St. ALOHA | | 9 | 250 | MOCNESS | D |
| *Janicella spinicauda* | 2007 | 4 | Sp | Cross Seamount | | 0 | 133 | Cobb Trawl | N |
| *Janicella spinicauda* | 2007 | 4 | Sp | Cross Seamount | | 0 | 133 | Cobb Trawl | N |
| *Janicella spinicauda* | 2007 | 4 | Sp | Cross Seamount | | 0 | 133 | Cobb Trawl | N |
| *Janicella spinicauda* | 2007 | 4 | Sp | Cross Seamount | | 0 | 133 | Cobb Trawl | N |
| *Janicella spinicauda* | 2007 | 4 | Sp | Cross Seamount | | 0 | 133 | Cobb Trawl | N |
| *Janicella spinicauda* | 2007 | 4 | Sp | Cross Seamount | | 0 | 195 | Cobb Trawl | N |
| *Janicella spinicauda* | 2007 | 4 | Sp | Cross Seamount | | 0 | 195 | Cobb Trawl | N |
| *Janicella spinicauda* | 2007 | 4 | Sp | Cross Seamount | | 0 | 195 | Cobb Trawl | N |
| *Janicella spinicauda* | 2007 | 4 | Sp | Cross Seamount | | 0 | 195 | Cobb Trawl | N |
| *Janicella spinicauda* | 2011 | 8 | S | St. ALOHA | | 398 | 596 | MOCNESS | D |
| *Japetella diaphana* | 2011 | 8 | S | St. ALOHA | | 1000 | 1500 | MOCNESS | N |
| *Japetella diaphana* | 2011 | 3 | Sp | W. Oahu | | 0 | 1000 | Tucker | N |
| *Japetella diaphana* | 2011 | 8 | S | W. Oahu | | 998 | 1251 | MOCNESS | N |
| *Lampanyctus nobilis* | 2010 | 5 | Sp | W. Oahu | | 0 | 250 | IKMT | N |
| *Lampanyctus nobilis* | 2008 | 4 | Sp | Cross Seamount | | 0 | 155 | Cobb Trawl | N |
| *Lampanyctus nobilis* | 2008 | 4 | Sp | Cross Seamount | | 0 | 155 | Cobb Trawl | N |
| *Lampanyctus nobilis* | 2011 | 3 | Sp | W. Oahu | | 0 | 250 | Tucker | N |
| *Lampanyctus nobilis* | 2011 | 3 | Sp | W. Oahu | | 0 | 800 | Tucker | N |
| *Lampanyctus nobilis* | 2008 | 4 | Sp | Cross Seamount | | 0 | 155 | Cobb Trawl | N |
| *Lampanyctus nobilis* | 2008 | 4 | Sp | Cross Seamount | | 0 | 155 | Cobb Trawl | N |
| *Lampanyctus nobilis* | 2008 | 4 | Sp | Cross Seamount | | 0 | 155 | Cobb Trawl | N |
| *Lampanyctus nobilis* | 2008 | 4 | Sp | Cross Seamount | | 0 | 155 | Cobb Trawl | N |
| *Liocranchia valdiviae* | 2011 | 3 | Sp | W. Oahu | | 0 | 800 | Tucker | N |
| *Liocranchia valdiviae* | 2010 | 5 | Sp | St. ALOHA | | 0 | 700 | IKMT | N |
| *Melanocetus johnsonii* | 2011 | 3 | Sp | W. Oahu | | 0 | 1500 | Tucker | N |
| *Melanocetus johnsonii* | 2011 | 3 | Sp | W. Oahu | | 0 | 700 | Tucker | D |
| *Myctophum lychnobium* | 2011 | 8 | S | St. ALOHA | | 596 | 796 | MOCNESS | D |
| *Myctophum lychnobium* | 2011 | 8 | S | St. ALOHA | | 0 | 10 | MOCNESS | D |
| *Nannobrachium nigrum* | 2011 | 3 | Sp | W. Oahu | | 0 | 250 | Tucker | N |
| *Nannobrachium nigrum* | 2011 | 3 | Sp | W. Oahu | | 0 | 700 | Tucker | D |
| *Nannobrachium nigrum* | 2011 | 8 | S | St. ALOHA | | 250 | 499 | MOCNESS | D |
| *Nannobrachium nigrum* | 2011 | 3 | Sp | W. Oahu | | 0 | 800 | Tucker | N |
| *Nannobrachium nigrum* | 2008 | 4 | Sp | Cross Seamount | | 0 | 210 | Cobb Trawl | N |
| *Nannobrachium nigrum* | 2008 | 4 | Sp | Cross Seamount | | 0 | 210 | Cobb Trawl | N |
| *Nannobrachium nigrum* | 2011 | 8 | S | St. ALOHA | | 9 | 250 | MOCNESS | D |
| *Nannobrachium nigrum* | 2008 | 4 | Sp | Cross Seamount | | 0 | 210 | Cobb Trawl | N |
| *Nannobrachium nigrum* | 2011 | 3 | Sp | W. Oahu | | 0 | 800 | Tucker | N |
| *Nannobrachium nigrum* | 2011 | 3 | Sp | W. Oahu | | 0 | 250 | Tucker | N |
| *Nannobrachium nigrum* | 2008 | 4 | Sp | Cross Seamount | | 0 | 210 | Cobb Trawl | N |
| *Nannobrachium nigrum* | 2008 | 4 | Sp | Cross Seamount | | 0 | 210 | Cobb Trawl | N |
| *Nannobrachium nigrum* | 2011 | 3 | Sp | W. Oahu | | 0 | 800 | Tucker | N |
| *Nannobrachium nigrum* | 2008 | 4 | Sp | Cross Seamount | | 0 | 210 | Cobb Trawl | N |
| *Nealotus tripes* | 2008 | 5 | Sp | Cross Seamount | | 0 | 202 | Cobb Trawl | N |
| *Nealotus tripes* | 2008 | 4 | Sp | Cross Seamount | | 0 | 197,8 | Cobb Trawl | N |
| *Nealotus tripes* | 2008 | 5 | Sp | Cross Seamount | | 0 | 202 | Cobb Trawl | N |
| *Nealotus tripes* | 2007 | 5 | Sp | Cross Seamount | | 0 | 184 | Cobb Trawl | N |
| *Nealotus tripes* | 2007 | 5 | Sp | Cross Seamount | | 0 | 184 | Cobb Trawl | N |
| *Nealotus tripes* | 2007 | 5 | Sp | Cross Seamount | | 0 | 184 | Cobb Trawl | N |
| *Nealotus tripes* | 2007 | 5 | Sp | Cross Seamount | | 0 | 184 | Cobb Trawl | N |
| *Nealotus tripes* | 2007 | 5 | Sp | Cross Seamount | | 0 | 184 | Cobb Trawl | N |
| *Notostomus gibbosus* | 2011 | 8 | S | St. ALOHA | | 498 | 1000 | MOCNESS | N |
| *Notostomus gibbosus* | 2011 | 8 | S | St. ALOHA | | 796 | 999 | MOCNESS | D |
| *Notostomus gibbosus* | 2011 | 3 | Sp | W. Oahu | | 0 | 1500 | Tucker | N |
| *Notostomus gibbosus* | 2011 | 8 | S | St. ALOHA | | 1500 | 2001 | MOCNESS | N |
| *Opisthoproctus soleatus* | 2011 | 8 | S | St. ALOHA | | 398 | 596 | MOCNESS | D |
| *Opisthoproctus soleatus* | 2011 | 8 | S | St. ALOHA | | 498 | 1000 | MOCNESS | N |
| *Opisthoproctus soleatus* | 2011 | 8 | S | St. ALOHA | | 0 | 0 | MOCNESS | D |
| *Opisthoproctus soleatus* | 2011 | 8 | S | St. ALOHA | | 498 | 1000 | MOCNESS | N |
| *Opisthoproctus soleatus* | 2011 | 8 | S | St. ALOHA | | 0 | 1502 | MOCNESS | D |
| *Oplophorus gracilirostris* | 2008 | 4 | Sp | Cross Seamount | | 0 | 503 | Cobb Trawl | D |
| *Oplophorus gracilirostris* | 2011 | 3 | Sp | W. Oahu | | 0 | 800 | Tucker | D |
| *Oplophorus gracilirostris* | 2008 | 4 | Sp | Cross Seamount | | 0 | 503 | Cobb Trawl | D |
| *Oplophorus gracilirostris* | 2008 | 4 | Sp | Cross Seamount | | 0 | 503 | Cobb Trawl | D |
| *Oplophorus gracilirostris* | 2011 | 3 | Sp | W. Oahu | | 0 | 800 | Tucker | D |
| *Oplophorus gracilirostris* | 2011 | 3 | Sp | W. Oahu | | 0 | 800 | Tucker | D |
| *Oplophorus gracilirostris* | 2008 | 4 | Sp | Cross Seamount | | 0 | 503 | Cobb Trawl | D |
| *Oplophorus gracilirostris* | 2011 | 8 | S | W. Oahu | | 2 | 248 | MOCNESS | N |
| *Oplophorus gracilirostris* | 2007 | 4 | Sp | Cross Seamount | | 0 | 626 | Cobb Trawl | D |
| *Oplophorus gracilirostris* | 2011 | 8 | S | W. Oahu | | 2 | 248 | MOCNESS | N |
| *Oplophorus gracilirostris* | 2007 | 4 | Sp | Cross Seamount | | 0 | 626 | Cobb Trawl | D |
| *Oplophorus gracilirostris* | 2007 | 4 | Sp | Cross Seamount | | 0 | 626 | Cobb Trawl | D |
| *Oplophorus gracilirostris* | 2014 | 2 | W | St. ALOHA | | 500 | 700 | MOCNESS | D |
| *Oplophorus gracilirostris* | 2014 | 2 | W | St. ALOHA | | 500 | 700 | MOCNESS | N |
| *Pterygioteuthis microlampas* | 2011 | 8 | S | St. ALOHA | | 9 | 250 | MOCNESS | D |
| *Pterygioteuthis microlampas* | 2011 | 8 | S | St. ALOHA | | 9 | 250 | MOCNESS | D |
| *Pterygioteuthis microlampas* | 2011 | 8 | S | St. ALOHA | | 9 | 250 | MOCNESS | D |
| *Pterygioteuthis microlampas* | 2011 | 8 | S | W. Oahu | | 2 | 248 | MOCNESS | N |
| *Pterygioteuthis microlampas* | 2011 | 3 | Sp | W. Oahu | | 0 | 800 | Tucker | N |
| *Pterygioteuthis microlampas* | 2011 | 3 | Sp | W. Oahu | | 0 | 800 | Tucker | D |
| *Sergestes erectus* | 2011 | 8 | S | St. ALOHA | | 9 | 250 | MOCNESS | D |
| *Sergestes erectus* | 2011 | 3 | Sp | W. Oahu | | 0 | 1000 | Tucker | N |
| *Sergestes erectus* | 2011 | 3 | Sp | W. Oahu | | 0 | 1000 | Tucker | N |
| *Sergestes erectus* | 2011 | 8 | S | St. ALOHA | | 1000 | 1502 | MOCNESS | N |
| *Sergestes erectus* | 2011 | 8 | S | St. ALOHA | | 9 | 250 | MOCNESS | D |
| *Sergestes erectus* | 2011 | 8 | S | W. Oahu | | 248 | 498 | MOCNESS | N |
| *Sergestes erectus* | 2011 | 3 | Sp | W. Oahu | | 0 | 800 | Tucker | D |
| *Sergia gardineri* | 2011 | 8 | S | St. ALOHA | | 1000 | 1500 | MOCNESS | N |
| *Sergia gardineri* | 2011 | 8 | S | St. ALOHA | | 498 | 1000 | MOCNESS | N |
| *Sergia gardineri* | 2011 | 8 | S | St. ALOHA | | 1000 | 1501 | MOCNESS | D |
| *Sergia gardineri* | 2011 | 8 | S | W. Oahu | | 701 | 799 | MOCNESS | D |
| *Sergia gardineri* | 2011 | 8 | S | W. Oahu | | 701 | 799 | MOCNESS | D |
| *Serrivomer sector* | 2011 | 8 | S | W. Oahu | | 498 | 751 | MOCNESS | N |
| *Serrivomer sector* | 2011 | 8 | S | St. ALOHA | | 796 | 999 | MOCNESS | D |
| *Serrivomer sector* | 2011 | 3 | Sp | W. Oahu | | 0 | 800 | Tucker | D |
| *Serrivomer sector* | 2011 | 3 | Sp | W. Oahu | | 0 | 800 | Tucker | N |
| *Serrivomer sector* | 2011 | 3 | Sp | W. Oahu | | 0 | 1500 | Tucker | N |
| *Serrivomer sector* | 2011 | 3 | Sp | W. Oahu | | 0 | 800 | Tucker | D |
| *Serrivomer sector* | 2011 | 3 | Sp | W. Oahu | | 0 | 700 | Tucker | D |
| *Serrivomer sector* | 2011 | 8 | S | St. ALOHA | | 596 | 796 | MOCNESS | D |
| *Sternoptyx pseudobscura* | 2010 | 5 | Sp | W. Oahu | | 0 | 300 | Ring Net |  |
| Stomatopoda | 2011 | 8 | S | St. ALOHA | | 2 | 247 | MOCNESS | N |
| Stomatopoda | 2011 | 3 | Sp | W. Oahu | | 0 | 800 | Tucker | D |
| Stomatopoda | 2011 | 8 | S | St. ALOHA | | 2 | 247 | MOCNESS | N |
| Stomatopoda | 2011 | 8 | S | St. ALOHA | | 0 | 0 | MOCNESS | D |
| Stomatopoda | 2011 | 3 | Sp | W. Oahu | | 0 | 800 | Tucker | N |
| Stomatopoda | 2011 | 3 | Sp | W. Oahu | | 0 | 800 | Tucker | D |
| Stomatopoda | 2007 | 4 | Sp | Cross Seamount | | 0 | 133 | Cobb Trawl | N |
| Stomatopoda | 2007 | 4 | Sp | Cross Seamount | | 0 | 133 | Cobb Trawl | N |
| *Systellaspis debilis* | 2011 | 8 | S | St. ALOHA | | 498 | 1000 | MOCNESS | N |
| *Systellaspis debilis* | 2011 | 8 | S | St. ALOHA | | 2 | 247 | MOCNESS | N |
| *Systellaspis debilis* | 2011 | 8 | S | St. ALOHA | | 596 | 796 | MOCNESS | D |
| *Vampyroteuthis infernalis* | 2011 | 3 | Sp | W. Oahu | | 0 | 800 | Tucker | D |
| *Vinciguerria* sp. | 2007 | 5 | Sp | Cross Seamount | | 0 | 183 | Cobb Trawl | N |
| *Vinciguerria* sp*.* | 2007 | 5 | Sp | Cross Seamount | | 0 | 183 | Cobb Trawl | N |
| *Vinciguerria* sp. | 2007 | 5 | Sp | Cross Seamount | | 0 | 183 | Cobb Trawl | N |
| *Vinciguerria* sp. | 2007 | 5 | Sp | Cross Seamount | | 0 | 183 | Cobb Trawl | N |
| *Vinciguerria* sp. | 2007 | 5 | Sp | Cross Seamount | | 0 | 183 | Cobb Trawl | N |

Table S2. Micronekton species reference for day and night depth ranges of occurrence and regression coefficients (a, b and *R^2^*) for linear regression of length (L, mm) against weight (W, g) used for specimens not weighted. All regression equations are based on the form: log_10_ (W) = a + b log_10_ (L).

| **Species** | **Depth ref.** | **Size type** | **Conversion factor** | **a** | **b** | ***R^2^*** |
| --- | --- | --- | --- | --- | --- | --- |
| *Abralia trigonura* | 1 |  |  |  |  |  |
| *Abraliopsis* sp*. A* | 1 |  |  |  |  |  |
| *Acanthephyra curtirostris* | 2 |  |  |  |  |  |
| *Argyropelecus hemigymnus* | 3 |  |  |  |  |  |
| *Argyropelecus sladeni* | 3 |  |  |  |  |  |
| *Benthosema suborbitale* | 4 |  |  |  |  |  |
| *Bolinichthys distofax* | 4 |  |  |  |  |  |
| *Bolinichthys longipes* | 4 | SL | This study | -4.824 | 2.969 | 0.92 |
| *Ceratoscopelus warmingii* | 5 |  |  |  |  |  |
| *Chauliodus sloani* | 6 | SL | This study | -5.852 | 3.113 | 0.97 |
| *Cyclothone alba* | 7 |  |  |  |  |  |
| *Cyclothone pallida* | 7 | SL | This study | -5.010 | 2.768 | 0.83 |
| *Cyema atrum* | 3 |  |  |  |  |  |
| *Diaphus perspicillatus* | 4 |  |  |  |  |  |
| Euphausiidae | 8 | CL | 18 |  |  |  |
| *Eurypharynx pelecanoides* | 9 |  |  |  |  |  |
| *Eustomias bifilis* | 6 |  |  |  |  |  |
| *Exocoetus volitans* | 10 |  |  |  |  |  |
| *Gempylus serpens* | 11 |  |  |  |  |  |
| *Gennadas bouvieri* | 12 |  |  |  |  |  |
| *Gnathophausia ingens* | 13 |  |  |  |  |  |
| *Gonostoma atlanticum* | 6 |  |  |  |  |  |
| *Hyaloteuthis pelagica* | 14 |  |  |  |  |  |
| *Hygophum proximum* | 4 | SL | This study | -5.226 | 3.239 | 0.99 |
| *Idiacanthus fasciola* | 6 | TL | This study | -4.016 | 1.892 | 0.91 |
| *Janicella spinicauda* | 2 |  |  |  |  |  |
| *Japetella diaphana* | 1 |  |  |  |  |  |
| *Lampanyctus nobilis* | 4 |  |  |  |  |  |
| *Liocranchia valdiviae* | 1 |  |  |  |  |  |
| *Melanocetus johnsonii* | 3 |  |  |  |  |  |
| *Myctophum lychnobium* | 4 |  |  |  |  |  |
| *Nannobrachium nigrum* | 4 |  |  |  |  |  |
| *Nealotus tripes* | 9 |  |  |  |  |  |
| *Notostomus gibbosus* | 2 |  |  |  |  |  |
| *Opisthoproctus soleatus* | 9 | SL | This study | -4.096 | 2.800 | 0.99 |
| *Oplophorus gracilirostris* | 13 | CL | This study | -3.769 | 3.161 | 0.74 |
| *Pterygioteuthis microlampas* | 1 |  |  |  |  |  |
| *Sergestes erectus* | 15 |  |  |  |  |  |
| *Sergia gardineri* | 15 |  |  |  |  |  |
| *Serrivomer sector* | 3 |  |  |  |  |  |
| *Sternoptyx pseudobscura* | 3 |  |  |  |  |  |
| Stomatopoda | 16 |  |  |  |  |  |
| *Systellaspis debilis* | 17 |  |  |  |  |  |
| *Vampyroteuthis infernalis* | 1 |  |  |  |  |  |
| *Vinciguerria* sp. | 6 |  |  |  |  |  |

|  |
| --- |

1. Young, R. E. 1978 Vertical distribution and photosensitive vesicles of pelagic cephalopods from hawaiian waters. Fish. Bull. 76, 583–615.
2. Podeswa, S. 2012. Active carbon transport and feeding ecology of pelagic decapods in the North Pacific Subtropical Gyre. Master thesis. Univ. of British Columbia.
3. Amesbury S. S. 1975. The vertical structure of midwater fish community off leeward Oahu, Hawaii. Ph.D. thesis. Univ. of Hawaii.
4. Clarke, T. A. 1973. Some aspects of the ecology of lanternfishes (myctophidae) in the Pacific Ocean near Hawaii. Fish. Bull. 71, 401–434.
5. Clarke, T. A. 1977. Diel feeding patterns of 16 species of mesopelagic fishes from Hawaiian waters. Fish. Bull. 76, 000-3.
6. Clarke, T. A. 1974. Some aspects of ecology of stomiatoid fishes in Pacific Ocean near Hawaii. Fish. Bull. 72: 337–351.
7. Maynard, S. D. 1982. Aspects of the biology of the mesopelagic fishes of the genus *Cyclothone* (Pisces: Gonostomatidae) in Hawaiian waters. Ph.D. thesis. Univ. of Hawaii.
8. Steinberg, D.K, J. S. Cope, S. E. Wilson, T. Kobari. 2008. A comparison of mesopelagic mesozooplankton community structure in the subtropical and subarctic North Pacific Ocean. Deep. Res. Part II Top. Stud. Oceanogr. 55, 1615–1635.
9. Clarke, A., P. J. Wagner. 1976. Vertical Distribution and Other Aspects of the Ecology of. 74, 635–645.
10. Gorelova, T. A. 1980. The feeding of young flyingfishes of the family Exocoetidae and of the smallwing flyingfish, *Oxyporhamhus micropterus*, of the family Hermirhamphidae. J. Ichthyol. 20: 60–71.
11. Hawn, D., M. Seki. 2005. End of the line: using instrumented longline to study vertical habitat of pelagic fishes. PFRP Newsletter 10(3)1:2.
12. Hendrickx, M. E., F. D. Estrada-Navarrete. 1989. A checklist of the species of pelagic (Penaoidea and Caridean) from the eastern pacific, with notes on their geographic and depth distribution. Unam 30, 104–121.
13. Cowles, D. L., J. J. Childress, M. E. Wells. 1991. Metabolic rates of midwater crustaceans as a function of depth of occurrence off the Hawaii Islands: food availability as a selective factor? Mar. Biol. 110, 75–83.
14. Jereb, P., C. F. E. Roper. 2010. Cephalopods of the world: an annotated and illustrated catalogue of cephalopod species known to date. Volume 2. Myopsid and oegopsid squids. FAO Species Catalogue for Fishery Purposes No. 4.
15. Walters, J. F. 1976. Ecology of Hawaiian sergestid shrimps (Penaeidea: Sergestidae). Fishery Bulletin, United States 74: 799-836.
16. Gloeckler, K., C. A. Choy, C. C. S., Hannides, H. G., Close, E., Goetze, B. N. Popp, J. C. Drazen. 2018. Stable isotope analysis of micronekton around Hawaii reveals suspended particles are an important nutritional source in the lower mesopelagic and upper bathypelagic zones. Limnol. Oceanogr. 63, 1168–1180
17. Zieman, D. A. 1975. Patterns of vertical distribution, vertical migration, and reproduction in the Hawaiian mesopelagic shrimp of the family Oplophroidae. Ph.D. thesis, Univ. of Hawaii.
18. Färber-Lorda, J. 1994. Length-weight relationships and coefficient of condition of Euphausia superba and Thysanoessa macrura (Crustacea: Euphausiacea) in south west Indian Ocean during summer. Mar. Biol. 118, 645–650.
